# Library resources constraints, frustration, and user behavior: An empirical library operations study

**DOI:** 10.1002/brb3.3627

**Published:** 2024-07-15

**Authors:** Raphael Aryee, Evelyn Ogboo Apronti Tetteh

**Affiliations:** ^1^ Faculty of Business Administration Methodist University Ghana Accra Ghana; ^2^ Library, Methodist University Ghana Accra Ghana

**Keywords:** aggressive behaviors, book hide and seek, book stealing, frustration, library resources

## Abstract

**Purpose:**

The lack of requisite library resources has an enormous effect on academic life in most universities. While previous studies have suggested that the lack of resources such as textbooks affects academic success, this study seeks to provide empirical evidence on the chain effect of the lack of recommended textbooks in universities.

**Design/methodology/approach:**

The study uses a quantitative dataset from 636 students from five public universities in Ghana collected using well‐structured questionnaires. The study adopts exploratory factor analysis, confirmatory factor analysis, and partial least squares structural equation modeling (PLS‐SEM) to analyze the measurement and structural models.

**Findings:**

The study concludes that limited library resources (such as recommended textbooks) frustrate library users and eventually birth antisocial behaviors such as stealing, hiding, and eroding books (or pages).

**Originality/value:**

This study highlights the significance of providing adequate library resources. It also guides library managers, policymakers, and scholars to manage library resources effectively.

## Graphical abstract

This study explains the effects of limited library resources on behavior of library users. The study concludes that limited recommended textbooks frustrate library users with eventually birth antisocial behaviors such as book stealing, hiding books, and book (or page) erosion.

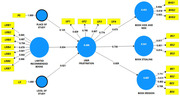

## 1 INTRODUCTION

A university library's operation is the hub of its intellectual life (Atuase and Filson, 2024; Farid et al., 2023; Scoulas & De Groote., 2023; Crawford, 2015). A library plays a critical role in the operations of every university by providing members of the university community (students, faculty, and administrative staff) with the requisite information and knowledge resources (Curran et al., 2006; Niziers and Richard, 2023; Saha and Roknuzzaman, 2024) for teaching, learning, and conducting research, which drives national development. Generally, most universities are interested in supporting the activities of their library since research performance is a crucial determinant of a university's reputation and financial success (Jubb, 2016; Romiani et al., 2024).

For any university library to effectively play its role, it must have the resources, that is, the textbooks and journals recommended by its faculty (Clamon et al., 2022; Scoulas and De Groote., 2023; Kapor and Weitzner, 2010). The lack of these resources can be detrimental to the success of any library (Appiah et al., 2024; Gkinni and Sarris, 2023; Dei and Asante 2022). This situation would eventually culminate in poor research, negatively affect knowledge generation, and eventually dent the university's image in terms of its rankings. Also, a library with its resources is essential for obtaining accreditation to run a tertiary institution in most countries (Etido and Wali, 2024; Mulimani and Naikar, 2024). Hence, the university risks being percieved as not plying its role in the knowledge ecosystem.

It is with no ambiguity to pontificate that all the 31,097 universities in the world, per the Ranking Web of Universities (2021), have a library that supports learning, teaching, and research. These libraries' fundamental objective is to provide users with relevant resources needed to generate knowledge (Atuase and Filson, 2024). However, owing to financial constraints on the part of the universities (Barsha and Munshi, 2024; Intahchomphoo et al., 2016; Crawford, 2015), most libraries cannot provide the requisite number of recommended textbooks that match the ratio of library users. This has undoubtedly led to inefficiency in library service (Baada et al., 2020) and struggle over the few resources. Intahchomphoo et al. (2016) conducted a comparative study on library funding in Canada and discovered that many libraries worldwide have dwindling resources. Even though, the student population of universities globally is increasing. A report by ICEF (2018) anticipated the global university student population to reach nearly 380 million by 2030, 472 million by 2035, and more than 594 million by 2040 (more than the population of the United States), from almost 216 million as of 2016. The issue of inadequate library resources is a challenge for many universities, specifically in Africa, and universities in Ghana are not exceptional.

The study was conducted in Ghana, a developing country in Africa that has earned the reputation as the center of quality education in Africa, with a foreign student population of 17,498 as of 2015, which is higher than most of her peers in the subregion (Statista, 2021). Indeed, according to the University World News (2022), Ghana is well‐positioned to attract students from all over Africa, the Caribbean, and other parts of the globe. Ghana currently has 15 public universities out of the 288 tertiary institutions in the country, with a student population of about 547,000 as of 2020 (Statista, 2021). Ghanaian public universities are known for their excellence in academia and research (Anane and Adusei, 2024; Gyamera and Asare, 2023). For example, a famous Ghanaian University was recently ranked fourth best in Africa and 24th in the world by the Times Higher Education World University ranking for 2023 (GhanaWeb, 2022). In 2022, the same university was ranked first worldwide by the same body for its research influence. The demand for these institutions is relatively high, especially with the introduction of the “free education” policy for secondary schools by the Government of Ghana.

Despite the expansion of public university education in Ghana, libraries in Ghana are under‐resourced (Agyen‐Gyasi and Atta‐Obeng, 2014; Ayiah and Tamakloe, 2023). This situation has led library users, especially students, to employ antisocial means to access library materials. In their studies on security and abuse of library materials within certain public universities in Ghana, Akussah and Bentil (2010) and Senyah (2004) and Udoudoh (2012) identify the scarcity of needed books and selfishness as the leading causes of book theft, mutilation, and book hiding among library users. Similarly, Appiah et al. (2024) revealed that some academic libraries in Ghana experienced unacceptable behavior due to inadequate library security personnel and inadequate library facilities. Because of this, this study joins the discourse by examining the chain effect of library materials (mainly textbooks) constraints on users' behavior using structural equation modeling(SEM) and primary empirical data from library users in public universities in Ghana.

## 2 LITERATURE REVIEW, THEORETICAL BACKGROUND, AND HYPOTHESES DEVELOPMENT

### 2.1 Library resources

Fundamentally, a resource has its roots in the resource‐based view theory, which argues that a firm is a bundle of heterogeneous resources and capabilities that support competitive advantage and explain the variance in performance across firms (Barney, 1991). From the viewpoint of library operations, resources refer to print and non‐printed materials in libraries that support curriculum activities such as learning, teaching, and research (Batchelor, 2017). These materials include newspapers, pamphlets, magazines, manuscripts, maps, documents, periodicals, cassettes, CDs, videotapes, DVDs, Blu‐ray discs, e‐books, audiobooks, journals, databases, and textbooks (Kaur Appiah et al., 2024 ; Bailey‐Hainer et al., 2014; Breeding, 2015; Scoulas, 2021). The faculty often recommend specific textbooks (Horsley et al., 2010) to support programs, predominantly undergraduate programs at university (Kapor and Weitzner, 2010).

Unfortunately, the cost of textbooks has been increasingly high. Popken (2015) noted that in the United States, the price of textbooks has quadrupled within 10 years at 1045% since 1977. Consequently, about 70%–75% of students cannot procure textbooks. For such students, the library is their last resort for recommended textbooks. Regrettably, the dwindling funds for libraries globally, coupled with increasing textbook prices, have significantly affected the provision of adequate textbooks (Jenkins et al., 2020; Todorinova & Wilkinson, 2019). Similarly, Nicholas et al. (2010) studied library operations and concluded that university libraries have financial constraints, affecting their acquisition of library resources. Moreover, Jubb (2010) discovered that the growing university population is increasing pressure on library materials. This situation has negatively impacted library services (Baada et al., 2020) and resulted in antisocial practices among library users (Akussah & Bentil, 2010; Senyah, 2004).

#### 2.1.1 Frustration and limited library resources

Frustration can be explained as irritable distress in response to limitation, exclusion, and failure (Jeronimus and Laceulle, 2020). It is a critical negative emotion that results from disappointment (Pyhäjärvi and Söderberg, 2024). It has been shown that frustration among library users results from the underperformance of university libraries. Saracevic et al. (1977) studied the causes and dynamics of user frustration in academic libraries and discovered that frustration results from poor library policy and operations. Frustration in this study is conceptualized as “(1) the nonfulfillment of an expected gratification, and (2) the instigation to aggression produced by a frustration is an inclination to hostile (or angry) and not instrumental aggression” (Berkowitz, 1988, p. 1). Library performance is determined to a large extent by the kind of resources or books that they have. Like others, library users will likely feel frustrated when their expectations of finding a book are unmet. Frustration manifests in several ways: anger, tension, annoyance, giving up, aggression, depression, and unhealthy behaviors. The frustration‐aggression theory well captures this effect. The reasoning behind this theory is that frustration in library settings is likely to birth unacceptable user behaviors such as pilfering or theft, mutilation, defacing of library material, hiding books in between shelves, keeping books beyond due dates, making noise, and loitering in library premises (Arinola et al., 2014; Isebe, 2014).

#### 2.1.2 User behavior

In this study, antisocial behavior is characterized as an overall lack of adherence to the social norms of socially acceptable behavioral patterns that can begin at any age, from adolescence to adulthood. Antisocial behaviors are common in libraries, and they include pilfering or theft, mutilation, defacing of library material, hiding books in between shelves, keeping books beyond due dates, chewing, eating, and drinking, noise‐making, littering, and loitering in library premises (Udoudoh 2012, Perez et al., 2009). Evidence in extant literature shows that the issue of book theft, hiding books within shelves, and mutilation is rampant (Isebe, 2014). These antisocial behaviors have been mainly associated with financial constraints. Jayasundara (2021) and Fasae and Adedokun (2016) blame the lack of funding for students in developing countries to purchase textbooks and other learning materials as causes of some anti‐social behaviours in libraries. i  Moreover, the phenomenon has also been associated with the lack of well‐planned user orientation (Adekunle et al., 2018) and poor library security systems (Innocent, 2019). Furthermore, ignorance and unavailability of photocopier services were identified as other factors responsible for these unacceptable behaviors among library users in some Nigerian universities (Arinola et al., 2014).

### 2.2 Theory of constraints and frustration‐aggression theory

According to Mentzer (2008), good research must be grounded in theory. Theories explain the relationship between variables or constructs in a model (Abend, 2008; Carter and Washispack, 2018). Because of the above, this study adopted two complementary theories: the theory of constraints (Goldratt and Cox, 1984) and the frustration‐aggression theory (Breuer & Elson, 2017). The theory of constraints is a management philosophy that focuses on the weakest ring(s) in the chain to improve the performance of systems (Zhao and Hou, 2022). It posits that every complex system consists of multiple linked activities, one of which constrains the entire system (Huang, Lu & Wang, 2021). This theory was adopted because it has been used in similar educational and service‐oriented studies such as Wuttor (2022), Bacelar‐Silva et al. (2024), and Kimani (2015). Also, the theory can explain the relationship between the constructs under study.

The frustration‐aggression theory is a seminal theory propounded by Dollard et al. (1939), which states that frustration precedes aggression, and aggression is the product of frustration (Breuer & Elson, 2017). This theory was employed because it has been used in other studies that sought to understand user behavior after being subject to the stimuli of frustration or conflict, such as Tade and Yikwabs (2020), Azemi, Ozuem and Howell (2020), and Battigalli et al. (2019). The triangulation of these theories helped the researchers sufficiently explain the predictive relationship between the constructs in the proposed research model and brought on board new perspectives, as suggested by Cairney (2013). This study used the theory of constraints and the frustration‐aggression theory to explain how students express their frustration over textbook constraints through antisocial behaviors in Ghanaian public universities.

### 2.3 Hypotheses development

**FIGURE 1:**
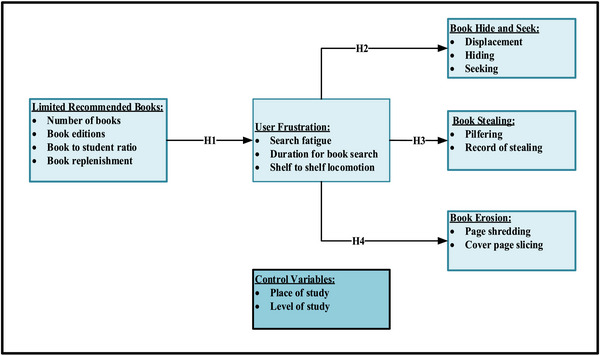
Conceptual framework of the study.

As demonstrated by the conceptual framework in Figure 1, this study consisted of five primary constructs: limited recommended textbooks, user frustrations, book hide and seek, stealing, and book mutilation/erosion. Limited recommended textbook is the exogenous construct; user frustration is the mediating construct; and the book hide and seek, stealing, and book mutilation/erosion are the endogenous constructs. It is obvious that limited recommended textbooks will frustrate library users. This logic is in line with the theory of constraints, which states that a particular weak link in a system can be a bottleneck in the entire system. That is, the lack of adequate recommended textbooks in the university library system can frustrate all facility users. Frustration in the system eventually makes users aggressive. This aggression is exhibited in negative attitudes such as hiding books, stealing, and tearing pages. This argument also aligns with the frustration‐aggression theory, which equivocally states that aggression is the product of frustration. Based on the above assertions, this study proposed four hypotheses as follows:

H1: Limited recommended books are positively related to user frustration.
H2: User frustration is positively related to book hide and seek.
H3: User frustration is positively related to book stealing.
H4: User frustration is positively related to book erosion.

## 3 RESEARCH METHODOLOGY

Like any empirical study, an appropriate methodological foundation is needed to derive a framework that shows the linkage between the various variables in library operations research. The selected methodology should promise objectivity and reproducibility. In line with the objective of this study, the quantitative research approach was adopted because the study seeks to understand the relationship between library resources, frustration, and user behavior variables. Using a well‐structured questionnaire, data were collected from 636 students from five public (government‐funded) universities in Ghana. This sample size was sufficient for the study because according to the G*Power software 3.1 (one of the modern ways of determining sample size), the minimum sample size for this study is 74, with a significant critical value of 1.66691. This sample size was determined using the *t*‐test family (and multiple linear regression: fixed model, single regression coefficient) functions, with an effect size *f* ^2^ of 0.15, *α err* of 0.05, power (1‐*β* err prob.) of 0.95, and three predictors (primary construct plus control variables) in a two‐tailed test.

Regarding sampling technique, these respondents were sampled via the convenience sampling approach because the researchers did not have access to a reliable sample frame. Nonetheless, because the convenience sampling technique is inherent with biases coupled with its lack of representativeness (Etikan et al., 2016), this study mitigated these defects by collecting data over 6 months from the students from the five targeted public universities. Finally, because the survey was online, to avoid duplication, students were questioned whether they had already filled out a similar questionnaire before being allowed to partake in the research. The five public universities were chosen because they were generally expected to have the required library resources, owing to the financial support they get from the government. However, the substantial student populations in these universities put pressure on these resources, birthing all kinds of frustration and behaviors. Therefore, the decision to conduct this study in the five populous public universities was not misplaced. After settling on the study context, the researchers sought permission from the relevant gatekeepers in these universities. After reviewing the content of the questionnaire, they all consented before the study was conducted. The respondents were further informed about the study's objectives and how their contributions will aid in conceptualizing the effect of limited library resources and user behavior in a developing country. Also, the respondents were informed that their participation was solely voluntary and that they had the right to opt out of the process at any time. Anonymity and confidentiality were guaranteed and observed. Finally, in line with the principle of research ethics, the data were collected during the free time of respondents, and the data were only used for the purpose for which it was collected. Table 1 summarizes the profile of the respondents surveyed. The questionnaires were administered online using Google Forms. The link to the Google form was shared with various students of these universities via their WhatsApp groups from April 15, 2022, to September 17, 2022.

**TABLE 1:** Sample profile.

Sample size (*n* = 636)	No. of occurrences	Percentage of occurrences
**Gender**		
Male	363	57.1
Female	273	42.9
**Age**		
17–24	316	49.7
25–32	199	31.3
33–40	62	9.7
41–50	34	5.3
50 and above	25	3.9
**Education**		
Undergraduate	445	66.2
Postgraduate	153	22.8
Others	38	5.7
**Area of study**		
Sciences	157	23.4
Business and Management	269	40.0
Humanities, Arts and others	210	31.3
**Campus of study**		
University A	247	38.8
University B	209	32.9
University C	120	18.9
University D	36	5.7
University E	24	3.8

Before the data were collected, the researchers authenticated the identity of the respondents as actual students of the universities mentioned above and assured respondents of the anonymity of their identity. The dataset was then exported from Excel to SPSS 20. Through online tools, respondents could not skip any question; hence, there were no missing data issues and, consequently, no need to correct missing data. The researchers then proceeded to examine nonresponse bias using the independent *t*‐test to compare the early and late responses for all the constructs in the model. The earlier respondents responded to the questionnaire from April 15 to June 15, and the late respondents responded from June 15 to September 17, 2022. The results revealed that the significance levels of the pairs were >0.05. Therefore, the study supported the null hypothesis, which says the true mean difference between the paired samples is zero, implying that nonresponse bias was not a problem in this study.

Moreover, since the same 7‐point Likert scale was used to collect data on all the constructs and from the same source at the same time, it prone the study to common method variance (CMV) (MacKenzie and Podsakoff, 2012), which tends to create bias parameter estimates and ultimately lead to wrong conclusions (Jakobsen and Jensen, 2015). As a result, this study used procedural and statistical approaches to mitigate CMV. The procedural method includes assuring informants of anonymity and explaining that there are no correct or incorrect answers to diminish respondents' reluctance to be surveyed. This made them less likely to provide socially desirable and consistent answers across questions (Podsakoff et al., 2003), for example, to reduce respondents who attach high importance to social desirability from overrating their library status. Finally, the length of the survey instrument was kept short (MacKenzie and Podsakoff, 2012) to reduce fatigue, which could affect the respondent's willingness to respond to the question accurately (MacKenzie and Podsakoff, 2012). Afterwards, the researchers adopted Harman's single‐factor test (during the exploratory factor analysis [EFA] phase of the study) to examine the effect of the CMV. All the items were loaded onto one common factor during this test using the principal axis extraction and no rotation approach (MacKenzie and Podsakoff, 2012). The total variance for the single factor extraction was 37.495%, less than 50%, suggesting that CMV did not affect the dataset used in this study.

### 3.1 Questionnaire design and measures used for the study

In line with recommendations from Saunders et al. (2016), this study adapted existing scales to assess the limited recommended textbooks, user frustrations, and stealing because these are well‐established constructs in extant literature. The limited recommended textbook construct was measured with seven indicators adapted from Hilton (2016) and Gurung and Martin (2011). User frustrations were measured using four items adapted from Tindall and Curtis (2019), Hadlington and Scase (2018), and Hazlett (2003). The book‐stealing construct was measured with three adapted items from Yousuf Ali (2017) and Mansfield (2009). However, because the book hide and seek and book erosion constructs were not well developed in the extant literature, this study developed scales for them. Hence, the book hide and seek construct had three items developed from studies by Abusin and Zainab (2010), Ajayi (2003), and Abifarin (1997). Finally, the book erosion construct was with three items developed from studies such as Raji et al. (2017), Olajide (2017), Lilly, Schloman and Hu (1991), and Hendrick and Murfin (1974). To further assess the survey instrument, the researcher subjected it to the scrutiny of academics and library experts and later pretested it with 50 respondents.

### 3.2 Control variables

To strengthen the validity of the conclusion in this study, the study sought to rule out any plausible alternative explanations. Hence, the statistical control technique was used, and data were collected on two variables expected to be both extraneous and influential to the research question (Kish, 2017). The two variables are the place of study and the level of study. The place of study was operationalized as the study's campuses. It was measured using 1 = University A students, 2 = University B students, 3 = University C, 4 = University D students, and 5 = University E students. The level of study was operationalized based on the kind of program being pursued. It was measured using 1 = undergraduate program, 2 = postgraduate program, and 3 = others.

### 3.3 Presentation of empirical results

#### 3.3.1 Reliability and validity

To get reliable results, this study evaluated the measurement model by determining its reliability and validity, as Hair et al. (2020) and Saunders et al. (2016) recommended. Cronbach's α and composite reliability (CR) were the criteria used to assess the measurement model owing to the use of reflective indicators. The results in Table 2 show that Cronbach's α values range from 0.772 to 1.000. These values exceed the threshold of 0.7 (Hair and Alamer, 2022; Ursachi et al., 2015). The CR values were from 0.866 to 1.000. Therefore, the scales used in this study were reliable. Moreover, it is essential to note that before the quality assessment, the unidimensionality of the items was evaluated first using EFA. EFA was performed using maximum likelihood analysis and Promax rotation with Kaiser normalizations to determine the factors. The outcome of this analysis showed construct unidimensionality, and the priori number of components was obtained. The Kaiser–Meyer–Olkin for sampling adequacy was 0.871. This outcome implied the sample size was adequate to perform the EFA. Besides, the χ‐square score of 16929.030 was obtained for the final EFA solution. Bartlett's test of sphericity was significant, with a *p*‐value less than 0.05. The commonalities produced were more than 0.5. All the components extracted had an eigenvalue of more than 1.00. The cumulative total variance explained of 73.802% (more than 50%) was obtained. This result shows that the factors retained accounted for 73.802% of the variations in the dataset.

**TABLE 2:** Measurement model.

Indicators	Contracts/item description	Loadings	Cronbach's α	Rho_A	Composite reliability	Average variance extracted (AVE)
	Limited recommended textbooks		0.859	0.872	0.892	0.544
LRB1	Our library lacks the recommended books that our lecturers prescribed	0.665				
LRB2	Our library lacks adequate number of recommended textbooks	0.687				
LRB3	Our library lacks sufficient copies of modern books	0.756				
LRB4	Our library's book to student ratio is poor	0.679				
LRB5	Our library lacks the current editions of recommended textbooks	0.841				
LRB6	Our library stocks the shelves with new books often	0.842				
LRB7	Our library has only one copy of our recommended books	0.666				
	**User frustration**		**0.815**	**0.817**	**0.878**	**0.643**
UF1	At our library, students will have to move from shelf to shelf several times in finding books	0.829				
UF2	At our library, sometimes, students can search for books and will never find it even if no one is using the book	0.795				
UF3	At our library, students are not able to borrow recommended textbooks	0.807				
UF4	At our library, students are not able to photocopy recommended textbooks	0.774				
	**Book hide and seek**		**0.776**	**0.806**	**0.877**	**0.711**
BHS1	At our library, students hide recommended textbooks	0.938				
BHS2	At our library students displace recommended books from their prescribed shelves	0.618				
BHS3	At our library, students have to search for books at their designated and undesignated shelves before they find them.	0.933				
	**Book stealing**		**0.772**	**0.778**	**0.867**	**0.685**
BS1	At our library, students steal recommended textbooks	0.817				
BS2	At our library, students have been caught stealing library books	0.851				
BS3	At our library, students have been caught stealing library books	0.814				
	**Book erosion**		**0.800**	**0.801**	**0.866**	**0.620**
BE1	At our library students deliberately tear cover pages	0.793				
BE2	At our library students deliberately tear cover pages	0.839				
BE3	At our library students have been caught with pages of their textbooks in their bags	0.802				
BE4	At our library students have to search for books a long time before they find it	0.709				
	**Control variables**					
PS	The university you attend	1.000	**1.000**	**1.000**	**1.000**	**1.000**
LS	Your current level of study	1.000	**1.000**	**1.000**	**1.000**	**1.000**

Abbreviations: BE, book erosion; BHS, book hide and seek; BS, book stealing; PS, place of study; LRB, limited recommended text books; LS, level of study UF, user frustration.

After proving the items' reliability, the next phase was to evaluate their validity. This was determined using four criteria: content (face), criterion (nomological), convergent, and discriminant validities. Face validity was achieved before data collection via previous literature, expert scrutiny, and piloting. Criterion validity was achieved due to the positive and significant correlations between the primary constructs (Boso et al., 2013). Also, convergent validity was achieved because the average variance extracted was >0.5 (Hair et al., 2020) and indicator loadings were >0.7, as stated by Hair et al. (2017), except for five items: LRB1, LRB2, LRB4, LRB7, and BHS2. However, since these items did not negatively affect the threshold of Cronbach's α, they were maintained (Hair et al., 2017). Besides, the discriminant validity was achieved using the indicator cross‐loadings, Fornell–Larcker criterion, and heterotrait‐monotrait (HTMT) ratio of correlations (Henseler et al., 2015). The indicator cross‐loadings (see Table 3) loaded more under their respective constructs than their cross‐loadings, in line with Hair et al. (2020). The results in Table 4 for the Fornell–Larcker show that the diagonals' correlations are greater than the inner correlations, except for book stealing and user frustration. Moreover, the HTMT values (Table 5) did not exceed 0.9, indicating that discriminant validity has been established, as Henseler et al. (2015) noted. These findings imply that this study met two of the three criteria for discriminant validity.

**TABLE 3:** Indicator cross‐loadings.

Items	Book erosion	Book hide and seek	Book stealing	Level of study	Limited recommended books	Place of study	User frustration
BE1	*0.793*	0.572	0.774	0.003	0.405	0.016	0.754
BE2	*0.839*	0.788	0.816	−0.004	0.480	−0.005	0.525
BE3	*0.802*	0.744	0.772	−0.017	0.435	−0.019	0.487
BE4	*0.709*	0.480	0.465	0.070	0.486	0.063	0.729
BHS1	0.797	*0.938*	0.820	−0.023	0.530	−0.008	0.612
BHS2	0.418	*0.618*	0.498	0.000	0.462	0.007	0.485
BHS3	0.763	*0.933*	0.803	0.003	0.530	0.021	0.591
BS1	0.687	0.599	*0.817*	−0.009	0.406	0.000	0.681
BS2	0.702	0.760	*0.851*	0.007	0.450	0.033	0.598
BS3	0.833	0.786	*0.814*	−0.001	0.481	−0.003	0.527
LS	0.022	−0.009	−0.002	*1.000*	−0.041	0.936	0.070
LRB1	0.387	0.420	0.382	−0.095	0.665	−0.083	0.421
LRB2	0.329	0.328	0.262	0.032	0.687	0.030	0.392
LRB3	0.555	0.550	0.504	0.044	*0.756*	0.034	0.576
LRB4	0.328	0.385	0.314	0.025	*0.679*	0.008	0.411
LRB5	0.494	0.520	0.465	−0.103	*0.841*	−0.113	0.513
LRB6	0.490	0.517	0.464	−0.111	*0.842*	−0.121	0.512
LRB7	0.320	0.335	0.298	0.012	*0.666*	−0.007	0.363
PS	0.023	0.008	0.012	0.936	−0.052	*1.000*	0.073
UF1	0.644	0.545	0.545	0.061	0.573	0.064	*0.829*
UF2	0.610	0.493	0.485	0.096	0.515	0.093	*0.795*
UF3	0.587	0.533	0.517	0.093	0.527	0.086	*0.807*
UF4	0.789	0.575	0.773	−0.012	0.411	0.001	*0.774*

*Note*: From the perspective of Chin (2009), the indicator allocated to a construct should load higher on that construct compared to others. To achieve discriminant validity the italic items loadings should exceed all others in the row.

**TABLE 4:** Discriminant validity (Fornell–Larcker criterion).

	Book erosion	Book hide and seek	Book stealing	Level of study	Limited recommended books	Place of study	User frustration
Book erosion	*0.787*						
Book hide and seek	0.801	*0.843*					
Book stealing	0.888	0.854	*0.828*				
Level of study	0.022	−0.009	−0.002	*1.000*			
Limited recommended books	0.577	0.605	0.535	−0.041	*0.737*		
Place of study	0.023	0.008	0.012	0.936	−0.052	*1.000*	
User frustration	0.829	0.673	0.736	0.07	0.628	0.073	0.802

*Note*: According to the Fornell–Larcker criterion, the values in the diagonals (in italics) should be more than those in the rows. The table indicates that these criteria was not met..

**TABLE 5:** Discriminant validity (Heterotrait‐monotrait [HTMT] ratio of correlations).

	Book erosion	Book hide and seek	Book stealing	Level of study	Limited recommended books	Place of study	User frustration
Book erosion							
Book hide and seek	0.825						
Book stealing	0.751	0.708					
Level of study	0.033	0.012	0.008				
Limited recommended books	0.673	0.734	0.646	0.089			
Place of study	0.037	0.016	0.017	0.736	0.083		
User frustration	0.567	0.846	0.802	0.09	0.744	0.084	

*Note*: According to Hair et al. (2017), the heterotrait‐monotrait (HTMT) ratio of correlations values should not exceed 0.9 suggests discriminant validity.

#### 3.3.2 Structural equation modeling

After proving the measurement model's adequacy, the structural model's veracity was ascertained using partial least squares structural equation modeling (PLS‐SEM). Unlike the covariance‐based SEM, the PLS‐SEM has a higher statistical power; hence, it is more sensitive to identifying significant relationships in a dataset (Hair et al., 2019). Nevertheless, before structural model analysis, this study checked if the dataset met the multivariate assumptions of normality, multicollinearity, and the presence of outliers (Gujarati, 2021; Williams et al., 2013). The Mardia's multivariate normality test proved the data were not normally distributed, and the thresholds for skewness and kurtosis were violated (see Table 6). The Mardia multivariate skewness and kurtosis threshold are ±1 or ±20, respectively (Wulandari et al., 2021). Another assumption of classical linear regression examined in this study was the absence of outliers because their presence distorts expected values (means), which is at the heart of regression analysis (Gujarati, 2021). In this study, Cook's distance technique was employed to determine the presence of outliers in the dataset. This method illustrates the degree to which regression coefficients change if a particular case is removed from a regression model (Inci et al., 2015). This technique is mainly used to determine the impact of a data point in ordinary least squares regression. A case with Cook's distance exceeding 1.0 may indicate an extraordinary influence (Inci et al., 2015; Kim & Storer, 1996). The test results were not >1.0, illustrating the absence of outliers in the dataset. This result shows that this research did not violate the assumption of the lack of outliers.

**TABLE 6:** Normality test results.

Mardia's multivariate skewness and kurtosis			
Sample size, *n* = 636			
Number of items = 23			
	*b*	*Z*	*p*‐value
Skewness	919.5032	97467.3365	0.000
Kurtosis	1731.4249	429.9986	0.000

Having satisfied most of the multivariate assumptions, the next stage was to proceed with the PLS‐SEM analysis. The structural integrity of the model was evaluated using the significance of path coefficients, the coefficients of determination *R^2^*, effect size *f*,*^2^* and predictive relevance *Q^2^* (Geisser, 1974; Hair et al., 2020). It is important to note that all path relationships with negative coefficients were insignificant. The results in Table 7 illustrate all the path confidence intervals obtained at a 95% confidence level and 5% significance level. The *R* ^2^ (limited recommended books and user frustration) is 40.6%. The *R* ^2^ (book hide and seek) is 45.3%, *R^2^* (book stealing) is 54.1%, and *R^2^* (book erosion) is 68.7%. This outcome shows a moderate variance explanation (Hair et al., 2017). Hair et al. (2017) suggested that *R* ^2^ values should be equal to or >10% for the variance explained to be deemed adequate. Besides, the *f* ^2^ (limited recommended books—user frustration) is 0.675; *f* ^2^ (user frustration—book hide and seek) is 0.827; f^2^ (user frustration—book stealing) is 1.179; and (user frustration—book erosion) is 2.193. From the standpoint of Cohen et al. (1998), the *f* ^2^ of 0.02 or more is small, 0.15 or more is medium, and 0.35 or more is large. This implies that the effect sizes in this study were large. Besides, *Q^2^* (user frustration) = 0.239; *Q^2^* (book hide and seek) = 0.244; *Q^2^* (book stealing) = 0.247, and *Q^2^* (book erosion) = 0.265. According to Hair et al. (2017), *Q^2^* must be greater than zero to establish a particular construct's predictive relevance or accuracy in the model. Using this criterion, it can be concluded that the endogenous constructs in the model have predictive relevance.

**TABLE 7:** Partial least squares structural equation modeling (PLS‐SEM) results.

Hypo.	Path relationship	Original sample	Standard deviation	*T* statistics	Decision	*p* values	2.50%	97.50%
	Level of study > user frustration	−0.021	0.101	0.207	Not supported	.836	−0.212	0.182
	Place of study > user frustration	0.125	0.057	2.193	Supported	.041	0.098	0.292
H1	Limited recommended books > user frustration	0.634	0.022	28.551	Supported	.000	0.590	0.673
H2	User frustration > book hide and seek	0.673	0.026	26.341	Supported	.000	0.623	0.717
H3	User frustration > book stealing	0.736	0.020	36.402	Supported	.000	0.695	0.771
H4	User frustration > book erosion	0.829	0.010	83.864	Supported	.000	0.810	0.849
	Model statistics							
	*R* ^2^ (user frustration)	0.406						
	Adjusted *R* ^2^ (user frustration)	0.403						
	*R* ^2^ (book hide and seek)	0.453						
	Adjusted *R* ^2^ (book hide and seek)	0.452						
	*R* ^2^ (book stealing)	0.541						
	Adjusted *R* ^2^ (book stealing)	0.540						
	*R* ^2^ (book erosion)	0.687						
	Adjusted *R* ^2^ (book erosion)	0.686						

**FIGURE 2:**
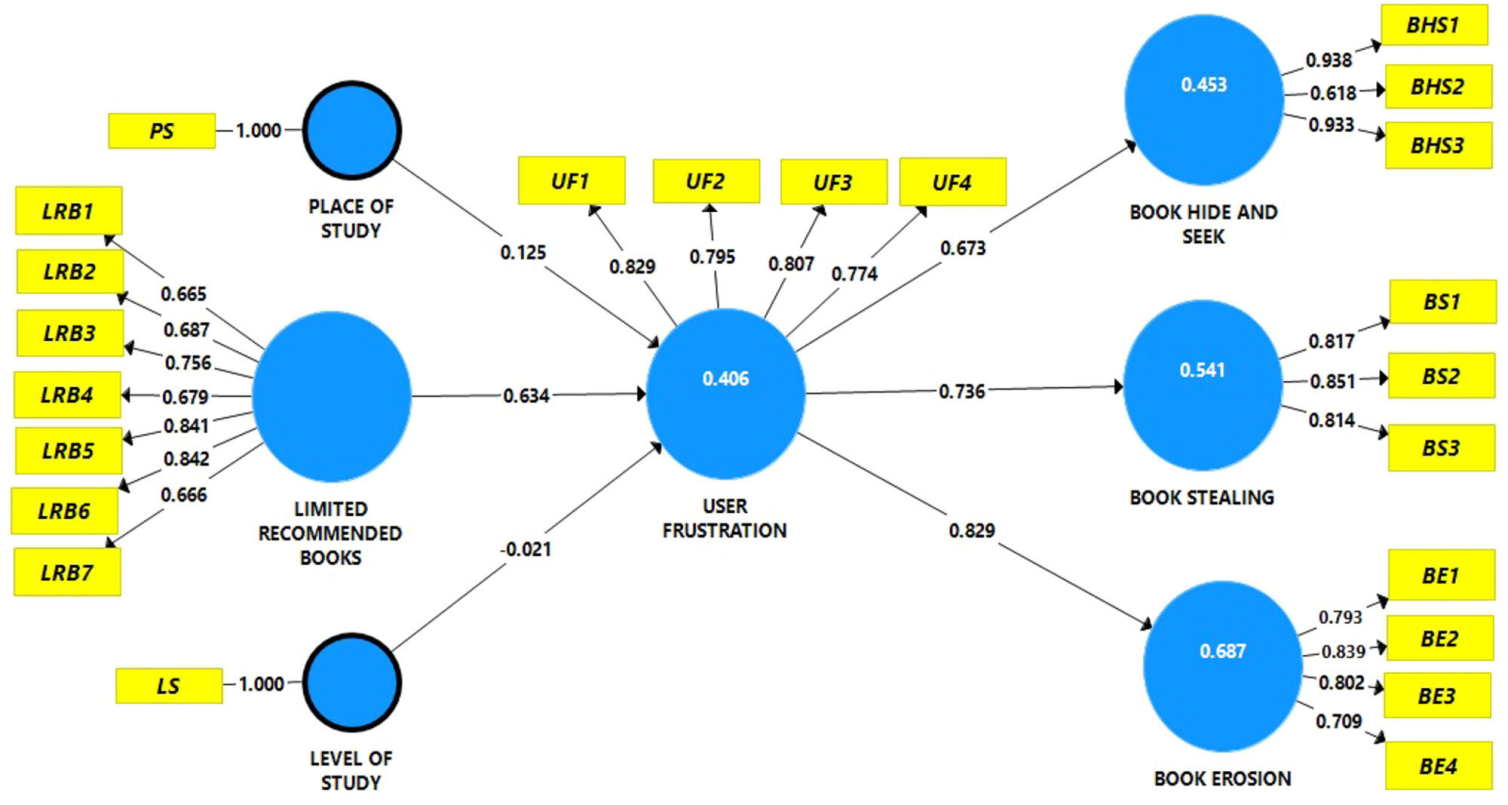
Partial least squares structural equation modeling (PLS‐SEM) path diagram. BE, book erosion; BHS, book hide and seek; BS, book stealing; PS, place of study; LRB, limited recommended text books; LS, level of study UF, user frustration.

Now that the model's explanatory and predictive powers have been substantiated, the study examined the relevance of the path coefficients. As displayed in Table 7, the control path results show that the place of study was significant (*β* = .125, *t*‐value = 2.193) with CI (confidence interval) from 0.098 to 0.292 (see Figure 2). In contrast, the level of study was found to be insignificant (*β* = −.021, *t*‐value = 0.207) with CI between −0.212 and 0.182. For the main paths, H_1_ dealt with the relationship between limited recommended books and user frustration, which was significant (*β* = .634, *t*‐value = 28.551) with CI between 0.59 and 0.673, and H_2_ dealt with the relationship between user frustration and book hide and seek. This relationship, too, was significant (*β* = .673, *t*‐value = 26.341) with CI between 0.623 and 0.717. H_3_ deals with the relationship between user frustration and book stealing. This nexus was significant (*β* = .736, *t*‐value = 36.402) with CI between 0.695 and 0.771. H_4_ deals with the relationship between user frustration and book erosion, which was also significant (*β* = .829, *t*‐value = 83.864) with CI between 0.81 and 0.849.

## 4 DISCUSSION

The study was set out to examine the relationship between limited library resources (specifically recommended textbooks), frustration, and antisocial behaviors. Using insight from two complementary theories, the theory of constraints and the frustration‐aggression theory, a model was proposed, and five hypotheses, H_1_, H_2_, H_3_ and H_4_. were formulated and tested. The first hypothesis (H_1_) proposed a positive and significant relationship between limited recommended books and user frustration. The second hypothesis (H_2_) stated that there is a positive and significant relationship between limited recommended books and book hide and seek. The third hypothesis (H_3_) stated that there is a positive and significant relationship between limited recommended books and book stealing. The fourth hypothesis (H_4_) stated that there is a positive and significant relationship between limited recommended books and book erosion/mutilation.

The study's results supported all five propositions, as shown in Table 7. That is, from the first hypothesis, the study established that the lack of recommended textbooks in public universities frustrates library users (in this case, university students). This result is in line with the theory of constraints, which posits that every complex system consists of multiple linked activities. If one activity is constrained, it affects the entire system (Huang, Lu & Dang, 2021). From the context of this study, it has been discovered that constraints in the library resources (limited recommended textbooks) in university libraries have the propensity to generate negative emotions (frustration) in library users. This finding is a significant contribution of this study to extant literature. It has been established that resource constraint is an antecedent of frustration or frustration as an outcome of library resource constraints. This study differs from previous studies that merely suggested that limited resources frustrate library users. This current study has extended the perimeters of knowledge in this domain by objectively proving, using empirical data, the effect of resource constraints on user frustrations.

Furthermore, the study supported H_2_. That is, the study illustrated that the frustration that emanates from the limited recommended textbooks leads to antisocial behaviors such as book hide and seek. In other words, the study found that public universities' lack of recommended textbooks forces students to engage in unacceptable social behaviors. For example, some students selfishly hide books on library shelves so they alone can use these books all the time. Therefore, it is expected to find an engineering textbook hidden on a religion shelf or a logistics book hidden on a linguistics bookshelf. To curtail these acts, librarians and attendants periodically conduct shelf‐to‐shelf inspections to ensure the books are rightly positioned at their designated places. The weed‐like audacity of the perpetrators of these acts and the determination of librarians to cure it makes books oscillate between library shelves. So today, the library attendant may rightly position an engineering book on its rightful shelf, and tomorrow, the book will be found on another shelf. Another significant contribution of this study is that it empirically established the relationship is resource constraints, book hiding, and book seeking in public libraries.

Additionally, this study found support for H_3_. Specifically, the study empirically established that the frustration that results from the limited library resources also leads to book stealing. Precisely, the study's results suggest that the lack of the requisite number of textbooks makes students steal the few copies that may be available. Indeed, Usman (2013) and Ferrinho et al. (2004) noted individuals sometimes steal for survival. Amid the scarce books, rational students (without moral values) will likely steal a library book, owing to their parochial interest. Unlike Arinola et al. (2014) and Jayasundara (2021), who noted that the inadequacy of library materials contributes to unacceptable behaviors among library users in some universities, this study has used objective data and rigorous methodology to illustrate that library resource constraints lead to frustration, which also leads to the stealing of books.

Furthermore, this study supported H_4_. The results show that the frustration resulting from the textbook shortage in university libraries compels some students to shred relevant book portions. Indeed, scholars like Sharma (2023) and Nzewi (2023) noted that mutilation is a widespread practice in university libraries. They will steal or shred some pages if they cannot hide or steal the entire book. The continual shredding of pages of a particular book leads to eroding the book. Specifically, Nzewi (2023) and Sharma (2023) noted this harmful act of page shredding in university libraries leads to the destruction of knowledge. This is because once a page is mutilated, it distorts the logic or the pattern of knowledge in a book, further worsening library resource constraints. Past studies, such as Akussah and Bentil (2010) and Senyah (2004), who asserted that scarcity of needed books and selfishness are the leading causes of book mutilation and book hiding among library users in some public universities in Ghana. This current study has tested and confirmed the empirical relationship between frustration and book mutilation (book erosion).

Finally, this study's results support the frustration‐aggression theory that suggests that frustration precedes aggression and that aggression is the product of frustration (Breuer & Elson, 2017). Additionally, the finding is consistent with the Azemi et al.'s (2020) study, which used the frustration‐aggression theory to investigate how customers expressed dissatisfaction by spreading negative feedback about poor services. As explained by Zhao and Hou (2022), the theory of constraint focuses on the weakest ring(s) in the chain to improve the performance of systems. In this case, limited recommended textbooks (the weakest ring) can be addressed to enhance library service performance. The results can, therefore, inform managerial decisions regarding library book acquisition, especially in developing countries. Consequently, stocking university libraries with the requisite quantity and quality of recommended books will mitigate the level of frustration, which will, by extension, lower the level of antisocial behavior at the university libraries.

## 5 CONCLUSION

This study examined the influence of limited recommended textbooks on user frustration and its impact on user behavior. Moreover, the study examined the indirect effect of user frustration on user behavior. Using perspectives from two complementary theories, the theory of constraints and frustration‐aggression theory, a model was formulated, and five hypotheses were developed and empirically tested with the aid of objective data collected from 636 respondents from five public universities. This data were analyzed with the aid of Smart PLS‐SEM after ensuring that the dataset met most assumptions of multivariate analysis. Finally, the study established that limited recommended textbooks caused user frustration, and this frustration produced behaviors such as tearing, hiding, and stealing of books in libraries.

### 5.1 Implications, limitations, and directions for future studies

This study proposes that effective libraries require the requisite resources. This study has implications for theory and practice (managers of libraries). From the theoretical perspective, this study contributes to the existing literature by empirically establishing the relationship between limited recommended textbooks, book hide and seek, book stealing, and book erosion. Besides, the theoretical contribution of this study lies in integrating two complementary theories to establish the relationship between the study's constructs. Moreover, our study has implications for the practice; in that university, decision‐makers should consider furnishing their libraries with adequate recommended textbooks especially e‐books to prevent or reduce antisocial behaviors in libraries.

This study is limited because it was restricted to only five public universities in Ghana. Hence, the results may not represent the situation in other tertiary institutions in Ghana. Future studies can replicate this study in private universities, which do not receive funds from the government, unlike public universities. Also, similar studies can be done in the future, using lectures or administrative staff as respondents. Our study did not consider e‐resources; future studies can look at this. Furthermore, using an online questionnaire prone the study to a biased sample (toward tech‐savvy and more WhatsApp users). This affects the generalizability of the results. Future studies may consider data collection methods that allow for generalizability. Also, this study's results cannot be generalized (on statistical grounds) due to convenience sampling. Future studies should consider more representative sampling approaches, such as simple random sampling, to achieve generalizability of results. Finally, this study controlled for the level of study and place of study; future studies should control for ethical morality and greed since these external variables have a nexus with the variables in the model.

## AUTHOR CONTRIBUTIONS

**Raphael Aryee**: Conceptualization; data collection; data analysis; investigation; methodology; project administration; supervision; validation; writing—original draft; writing—review and editing. **Evelyn Ogboo Apronti Tetteh**: Conceptualization; data collection; investigation; validation; writing—original draft; writing—review and editing.

## FUNDING INFORMATION

The authors received no financial support for the research, authorship, and/or publication of this article.

## CONFLICT OF INTEREST STATEMENT

The authors declare no conflicts of interest.

### PEER REVIEW

The peer review history for this article is available at https://publons.com/publon/10.1002/brb3.3627

## Data Availability

The data that support the findings of this study are available on request from the corresponding author.
